# The Obesity Paradox Predicts the Second Wave of COVID-19 to Be Severe in Western Countries

**DOI:** 10.3390/ijerph18031029

**Published:** 2021-01-25

**Authors:** Indrikis A. Krams, Priit Jõers, Severi Luoto, Giedrius Trakimas, Vilnis Lietuvietis, Ronalds Krams, Irena Kaminska, Markus J. Rantala, Tatjana Krama

**Affiliations:** 1 Department of Biotechnology, Daugavpils University, LV5401 Daugavpils, Latvia; ronalds.krams@student.emu.ee (R.K.); tatjana.krama@du.lv (T.K.); 2 Institute of Ecology and Earth Sciences, University of Tartu, EE51014 Tartu, Estonia; 3 Department of Zoology and Animal Ecology, Faculty of Biology, University of Latvia, LV1004 Riga, Latvia; 4 Institute of Molecular and Cell Biology, University of Tartu, EE51010 Tartu, Estonia; priit.joers@ut.ee; 5 School of Psychology, University of Auckland, 1142 Auckland, New Zealand; s.luoto@auckland.ac.nz; 6 Institute of Biosciences, Vilnius University, 10257 Vilnius, Lithuania; giedrius.trakimas@gf.vu.lt; 7 Department of Surgery, Riga Stradins University, LV1007 Riga, Latvia; vilnis.lietuvietis@icloud.com; 8 Riga East Clinical University Hospital, LV1010 Riga, Latvia; 9 Department of Anatomy and Physiology, Daugavpils University, LV5401 Daugavpils, Latvia; irena.kaminska@du.lv; 10 Department of Biology, Section of Ecology, University of Turku, FI-20014 Turku, Finland; mjranta@utu.fi

**Keywords:** COVID-19, visceral adipose tissue, systemic inflammation, SARS-CoV-2, ACE2, weight gain, second wave, Quarantine-15

## Abstract

While COVID-19 infection and mortality rates are soaring in Western countries, Southeast Asian countries have successfully avoided the second wave of the SARS-CoV-2 pandemic despite high population density. We provide a biochemical hypothesis for the connection between low COVID-19 incidence, mortality rates, and high visceral adiposity in Southeast Asian populations. The SARS-CoV-2 virus uses angiotensin-converting enzyme 2 (ACE2) as a gateway into the human body. Although the highest expression levels of ACE2 are found in people’s visceral adipose tissue in Southeast Asia, this does not necessarily make them vulnerable to COVID-19. Hypothetically, high levels of visceral adiposity cause systemic inflammation, thus decreasing the ACE2 amount on the surface of both visceral adipocytes and alveolar epithelial type 2 cells in the lungs. Extra weight gained during the pandemic is expected to increase visceral adipose tissue in Southeast Asians, further decreasing the ACE2 pool. In contrast, weight gain can increase local inflammation in fat depots in Western people, leading to worse COVID-related outcomes. Because of the biological mechanisms associated with fat accumulation, inflammation, and their differential expression in Southeast Asian and Western populations, the second wave of the pandemic may be more severe in Western countries, while Southeast Asians may benefit from their higher visceral fat depots.

## 1. Introduction

A second wave of the COVID-19 disease has rapidly been gaining momentum since early fall 2020. Although governments and health systems seem to be more prepared for preventing and handling the pandemic, outbreaks of COVID-19 intensify, and many parts of the Western world have experienced a full-blown second surge of the pandemic. Once again, Europe and the United States are the epicenters of the disease, with lockdown restrictions being reimplemented as the effects of the second wave become more severe. In contrast, China has avoided its second wave so far, and looks stable with its infection and mortality rates [1]. Moreover, the whole region of Southeast Asia and Oceania have been hit by the coronavirus pandemics much less than other parts of the world. Countries such as Taiwan and Vietnam have successfully avoided the second wave of the SARS-CoV-2 infection in spite of their high population density.

**Figure 1:**
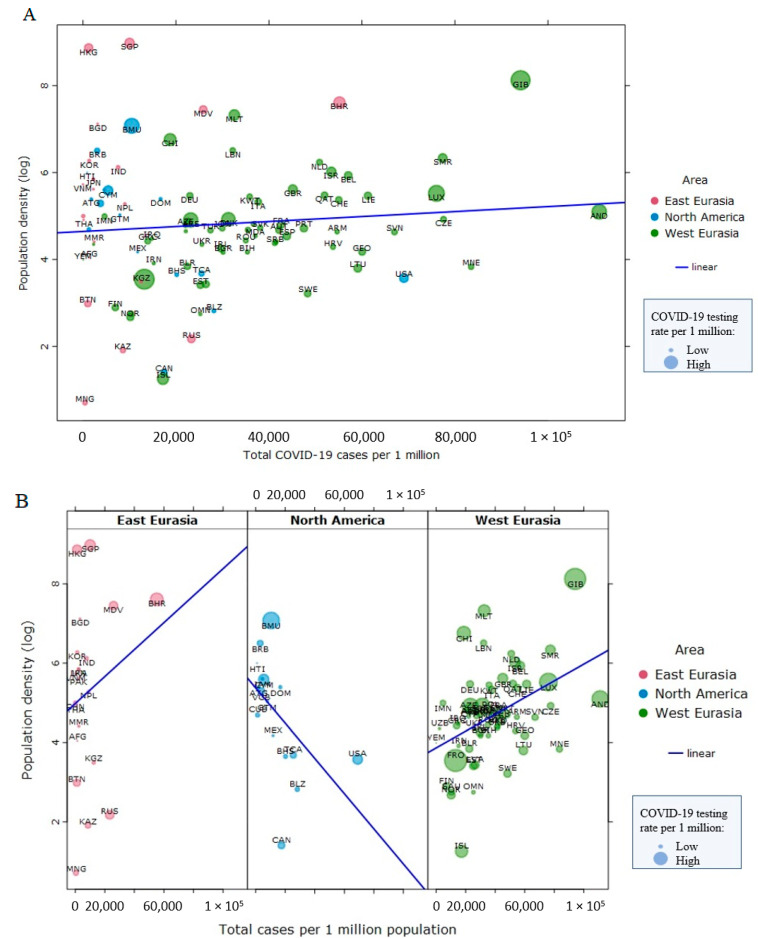
No relationship between population density and COVID-19 incidence (as of 10 January 2021) in three world regions combined (Panel **A**). Within three world regions, the relationships between population density and COVID-19 incidence go in the opposite directions (Panel **B**). Data points are scaled to reflect COVID-19 testing rates per 1 million population.

**Figure 2:**
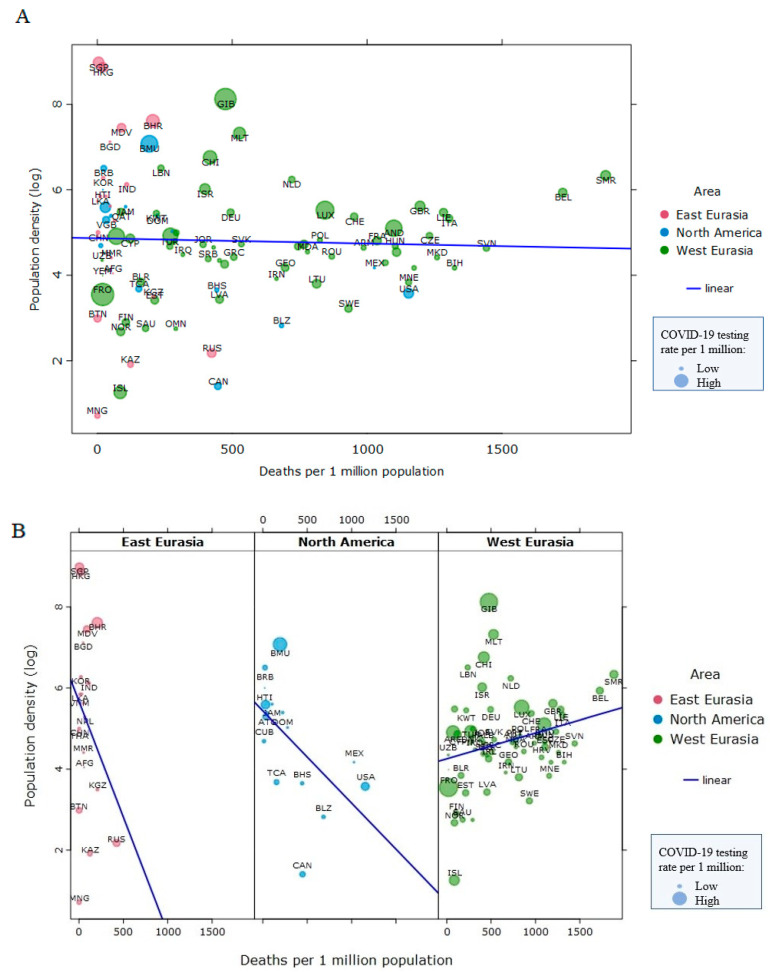
No relationship between population density and COVID-19 mortality (as of 10 January 2021) in three world regions combined (Panel **A**). Within three world regions, the relationships between population density and COVID-19 mortality go in the opposite directions (Panel **B**). Data points are scaled to reflect COVID-19 testing rates per 1 million population.

Overall, population density is not related to COVID-19 infection rates (*τ* = 0.02, *p* = 0.72, *n* = 99 nations, Figure 1A) nor to mortality rates cross-nationally in North America, East Eurasia, and West Eurasia (*τ* = −0.08, *p* = 0.24, *n* = 99 nations, Figure 2A), suggesting that factors other than population density play a more prominent role in the spread and severity of the COVID-19 disease [2]. Within world regions, population density was negatively associated with COVID-19 cases (per 1 million inhabitants) in North America (*τ* = −0.48, *p* = 0.009, *n* = 16), but positively in East Eurasia (*τ* = 0.13, *p* = 0.40, *n* = 21) and West Eurasia (*τ* = 0.29, *p* < 0.001, *n* = 62) (Figure 1B). Only in North America and West Eurasia were the associations statistically significant, though the effects were in opposing directions. We found that population density is not related to COVID-19 mortality (Figure 2A). Within world regions, population density was negatively associated with COVID-19 deaths (per 1 million inhabitants) in North America (*τ* = −0.47, *p* = 0.01, *n* = 16) and East Eurasia (*τ* = −0.03, *p* = 0.83, *n* = 21), but the association with COVID-19 deaths was positive in West Eurasia (*τ* = 0.16, *p* = 0.07, *n* = 62) (Figure 2B). Only in North America was the association statistically significant. These analyses were done in a sample of 99 countries from East Eurasia, North America, and West Eurasia, as those countries are the focus of the rest of this article. We used Kendall’s Tau to analyze these bivariate correlations because the data were non-normally distributed and had relatively small sample sizes. A larger sample of nations from all world regions yielded smaller, statistically non-significant correlations between population density (log) and cases per 1 million population (*r_s_* = 0.12, *p* = 0.11, *n* = 176), and between population density (log) and deaths per 1 million population (*r_s_* = 0.05, *p* = 0.53, *n* = 176). Data on total COVID-19 deaths and COVID-19 tests per 1 million inhabitants up to 10 January 2021 were collected from the Worldometer site (https://www.worldometers.info/coronavirus/#countries). Data on population density (2018) were collected from World Bank statistics. 

In this Perspective article, we argue why human populations differ in SARS-CoV-2 infection prevalence and severity based on their differential build-up of energy reserves into subcutaneous and visceral fat depots, and why the second wave of COVID-19 may be more severe in Western countries. While obesity is traditionally considered as a disorder of the energy homeostasis system, we suggest that certain types of fat may have a positive effect on COVID-19 infection rates and outcomes. This suggestion is based on the idea that organisms store body fat as a hedge against diseases to allow them to survive periods of pathogen-induced anorexia [3] and that visceral fat depots can help detect and eliminate pathogens and maintain immune homeostasis of the gut microbiome [4].

## 2. The Politics of Lockdown and the COVID-19 Infection

**Figure 3:**
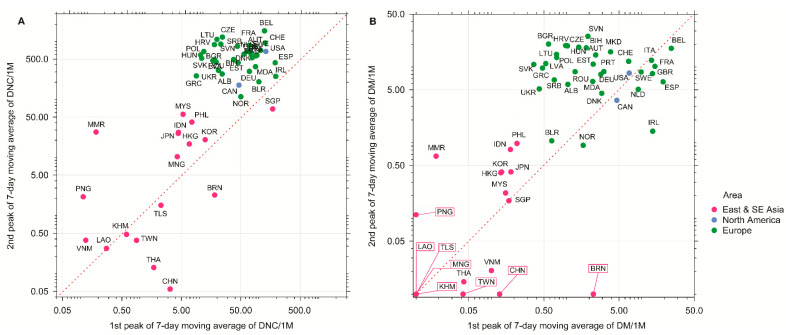
The peaks of (**A**) 7-day moving average of daily new cases per million population (DNC/1M); (**B**) 7-day moving average of daily mortality per million population (DM/1M) of East and South-east Asian countries (red dots), North American countries (blue dots), and European countries (green dots) on the log scale (as of 31 December 2020). Symbols above the diagonal (dashed line) indicate higher numbers in the second wave relative to the first wave, while symbols below the diagonal indicate the opposite. Framed labels indicate countries with zero mortality values on perpendicular axis.

Stringent quarantines, city lockdowns, local public health measures, and mandatory quarantines to ban or restrict international traffic and/or domestic traffic are considered the main reasons that stopped the SARS-CoV-2 virus’s spread in China, South Korea, and Singapore [5]. While Southeast Asian countries and New Zealand adopted the more stringent suppression strategy from the beginning of the pandemics [6], most Western countries (with the exception of the countries that were hit hardest during the first wave, such as Spain and Italy) initially focused on measures that only mitigated the spread of the virus and decreased transmission rate [5]. Although many European countries and the USA have started to follow more stringent suppression strategies since spring 2020, this change did not allow them to avoid the second wave of the pandemic, minimize mortalities, nor improve their economies. While Southeast Asian countries such as Vietnam, Laos, and Cambodia took serious measures to prevent the SARS-CoV-2 virus from spreading, they have never implemented the extreme quarantines characteristic of China and Italy. However, these countries have had consistently low incidence and mortality rates during the second wave (Figure 3), which is puzzling and requires further explanation. Understanding the success of these Southeast Asian countries is important, because the testing and especially production and implementation of effective vaccines against the SARS-CoV-2 virus may take more time than expected, increasing the death toll of COVID-19.

## 3. The Obesity Paradox, Visceral Fat, and COVID-19 Outcomes

Krams et al. (2020) [7] suggested that the obesity paradox provides a possible explanation for the observed population differences in COVID-19 infection and mortality rates between Europe, the USA, and Southeast Asia. In the obesity paradox, obese patients may have better health outcomes than normal-weight patients despite greater risk of local and systemic inflammation in their fat tissue [8]. Lower mortality in individuals with larger fat reserves has been reported in both infectious and non-communicable diseases [9,10,11,12]. The beneficial impact of body fat in multiple diseases is considered to provide a buffer against disease-induced anorexia, which is known to impair immunity and increase susceptibility to diseases and infections [3,13,14]. According to the obesity paradox hypothesis as applied to COVID-19, populations differ in their COVID-19 incidence and mortality rates because of the differences in the distribution and condition of adipose tissue [15]. Although adipose tissue is generally considered as an energy reservoir [3,15], subcutaneous fat (superficial subcutaneous adipose tissue and deep subcutaneous adipose tissue) and visceral fat, two main types of adipose tissue in the body, have important diverging metabolic and endocrinological roles [15,16,17,18]. Most body fat is subcutaneous, and it initially grows by hyperplasia (cell number increase). In obesity, the maximum amount of subcutaneous fat seems evolutionarily constrained [3]. At the maximum number of fat cells in the subcutaneous fat depot, the fat tissue becomes locally inflamed [19] and adipocyte hyperplasia normally stops. Fat tissue shifts its growth from hyperplasia to hypertrophy (cell size increases), leading to even higher local inflammation in subcutaneous adipocytes [19]. An increase in subcutaneous adipocyte hypertrophy [20] determines the onset of fat-storing in the visceral tissue depots. Visceral fat has long been known to be associated with systemic inflammation [21,22], insulin resistance, and other metabolic syndromes [16,17,23]. Visceral fat is harmful because it produces pro-inflammatory cytokines released directly into the bloodstream and can lead to auto-amplifying cytokine production called “cytokine storms” [24]. Therefore, reducing visceral fat and/or increasing its metabolic health is traditionally considered a preventive measure for metabolic diseases [24].

Surprisingly, visceral adiposity is considerably higher in Southeast Asian populations than in Europe or the USA [25], while COVID-19 incidence and mortality rates are higher in Western countries than in Southeast Asia (Figure 3A,B, respectively). In Southeast Asia, naturally occurring fat build-up involves building small subcutaneous fat depots and relatively large visceral adipose tissue depots [3,25,26]. Southeast Asians have greater body fat than Europeans and North Americans for the same BMI, meaning Southeast Asians cannot be treated as obese despite their larger visceral fat amounts [27]. It has been shown that the SARS-CoV-2 virus uses angiotensin-converting enzyme 2 (ACE2) as a gateway into the body [28,29,30]. ACE2 is a cell-surface exoenzyme that converts angiotensin II (Ang II) into vasodilatory angiotensin 1–7 (Ang 1–7). Importantly, adipose tissue, in general, has one of the highest expression levels of ACE2 of all tissues, being especially high in visceral adipose tissue [31]. Thus, ACE2 amount is expected to be highest in Southeast Asian populations that fare best in the COVID-19 pandemic [32,33]. However, it has been suggested that the ACE2 pool can be smaller in Southeast Asia than in Europe and North America [15]; otherwise, Southeast Asians would potentially host huge viral loads in their visceral adipose tissue, inducing more severe forms of the COVID-19 disease [34].

ACE2 function is known to be dysregulated in certain metabolic pathologies when removed from cells’ surface via cleavage by the transmembrane disintegrin and metalloproteinase 17 (ADAM17) [35]. Such shedding of ACE2 is frequently seen in inflammatory states associated with adiposity and obesity [32,33]. Therefore, the loss of ACE2 might explain the paradoxical connection between adipose tissue and COVID-19 infection. While visceral adipose tissue is considered to harbor a large pool of functional ACE2 molecules, the actual ACE2 amount in visceral adipose tissue of Southeast Asians may be much lower than that of Europeans and North Americans because of higher systemic inflammation in visceral fat in Southeast Asians [15]. This specific way of the visceral fat build-up of Southeast Asians might be caused by previous encounters with coronaviruses [36]. High levels of visceral adiposity can prevent the virus from entering human cells, creating the observed association between visceral adiposity and the mildest COVID-19 severity observed in Southeast Asia. Since the disruption of the molecular processes required for normal lung homeostasis starts within hours of the virus’s entrance into the body [37], preventing the virus from entering the cells is of paramount importance.

## 4. Pandemic-Induced Weight Gain and COVID-19

While city lockdowns and other restrictions are necessary measures to restrict the pandemics, the “Quarantine 15” (also known as the “Quarantine 19” in popular literature) has become a common way of referring to lockdown-induced weight gain during the COVID-19 pandemics [38,39]. Extra weight gained during lockdowns is caused by consuming more food than an individual can burn off through limited physical activity, especially when individuals consume energy-dense food which is poor in antioxidants and omega-3 fatty acids and rich in saturated fat and refined carbohydrates [40]. This so called “Westernized” diet and extra weight can affect the gut microbiome, resulting in changes in the host’s immune responses [41]. It has been also shown that consumption of energy-dense food poor in antioxidants and omega-3 fatty acids and rich in saturated fat and refined carbohydrates [40] may directly impair the host’s immune system [42]. Importantly, long-term lockdown and loneliness may cause people to eat foods known to cause obesity [43], which is associated with an increased risk of inflammation and mental health problems [44,45]. Therefore, phenomena like Quarantine 15 may have detrimental effects on people’s BMI, overall health, and, by extension, their ability to overcome COVID-19 [46,47,48].

Besides promoting unhealthy lifestyles, lockdown and lockdown-induced weight gain may directly affect immune response to viral infections through alterations of the cellular immune system [49]. Adiposity has been shown to decrease the strength of immune response to influenza and hepatitis-B vaccination [50,51,52] via impaired activation and fewer functional markers of blood mononuclear cells [49].

The effects caused by Quarantine 15 may differ between populations. We predict that a mechanism similar to the obesity paradox potentially alleviates the COVID-19 situation in Southeast Asia during the second wave [15,35,53]. A further increase in already high visceral fat levels in these populations will negatively affect the ACE2 pool not only in adipose tissue, but in all other parts of the body, including alveolar epithelial type 2 cells in the lungs due to visceral fat’s systemic effects operating via inflammation. Neuropilin-1 (NRP1) facilitates SARS-CoV-2 cell entry [54]; consequently, if there are no ACE2 receptors available on the surface of cells, as predicted by the reported high visceral fat levels in Southeast Asian populations, the potentiating factor effect of NRP1 is also expected to be absent, thus increasing protection against the SARS-CoV-2 virus. In contrast, in Europe and in the USA, where high visceral fat levels are less common than in Southeast Asia, people are expected to continue building all fat depots, including superficial and deep subcutaneous adipose tissues [53], causing mostly local adipocyte inflammation and not decreasing ACE2 levels throughout the organism. At the same time, weight gain causes deteriorating overall metabolic health and dysfunctional immune system, thereby decreasing an organism’s capability to withstand infections. Europeans, North Americans, and obese (because of their modern lifestyle) Southeast Asians are therefore expected to suffer from the second wave of the COVID-19 disease even more than during the first wave [55]. Thus, based on these hypothesized mechanisms, we predict that pandemic-induced weight gain has differential effects in different populations, generally beneficial in Southeast Asian populations and detrimental in Western populations [56,57].

## 5. Conclusions

We hope that conclusions drawn from the obesity paradox in the COVID-19 context may help governments and individuals properly prepare for 2021 until vaccines against SARS-CoV-2 become widely available, and when their efficacy has been properly verified in real-world scenarios taking into account immune function heterogeneity and different strains of COVID-19 at the level of individuals and populations. Several pharmaceutical companies have started the production of vaccines, and many countries have already started vaccinations at the beginning of 2021. However, the large-scale effectiveness, population vaccination rate, and length of acquired immunity particularly in the face of different COVID-19 strains are currently unknown. Since many parts of the world are in the midst of an overwhelming COVID-19 wave, it is crucial to pay attention to this physiological hypothesis to prevent unnecessary fatalities before sufficient vaccination can be acquired at the population level. The research reviewed here indicates that adipose tissue heterogeneity requires more attention in the COVID-19 context. We highlight that according to the obesity paradox, distinctive types of adipose tissue may have different roles in affecting COVID-19 infection rates and outcomes.
